# Lysates of *Lactobacillus acidophilus* combined with CTLA-4-blocking antibodies enhance antitumor immunity in a mouse colon cancer model

**DOI:** 10.1038/s41598-019-56661-y

**Published:** 2019-12-27

**Authors:** Qian Zhuo, Bohai Yu, Jing Zhou, Jingyun Zhang, Runling Zhang, Jingyan Xie, Qingling Wang, Shuli Zhao

**Affiliations:** 1 General Clinical Research Center, Nanjing First Hospital, Nanjing Medical University, Nanjing, 210006 China; 2 Department of Pathology, Xuzhou Medical University, Xuzhou, Jiangsu 221004 China; 3 Medical Laboratory Department, Shenzhen Hospital of Guangzhou University of Chinese Medicine, Shenzhen, Guangdong 518034 China; 4 University of Chinese Academy of Sciences Shenzhen Hospital, Shenzhen, Guangdong 518106 China

**Keywords:** Cancer prevention, Cancer immunotherapy

## Abstract

Previous reports have suggested that many gut microbiomes were associated with the development of colorectal cancer (CRC), and could modulate response to numerous forms of cancer therapy, including checkpoint blockade immunotherapy. Here we evaluated the protective efficacy of *Lactobacillus acidophilus (L. acidophilus)* cell lysates combined with an anti-CTL antigen-4 blocking antibody (CTLA-4 mAb) in syngeneic BALB/c mice CRC models induce by a single intraperitoneal injection of 10 mg/kg azoxymethane (AOM), followed by three cycles of 2% dextran sulfate sodium (DSS) in drinking water. In contrast to CTLA-4 mAb monotherapy, *L. acidophilus* lysates could attenuate the loss of body weight and the combined administration significantly protected mice against CRC development, which suggested that the lysates enhanced antitumor activity of CTLA-4 mAb in model mice. The enhanced efficacy was associated with the increased CD8 + T cell, increased effector memory T cells (CD44 + CD8 + CD62L+), decreased Treg (CD4 + CD25 + Foxp3+) and M2 macrophages (F4/80 + CD206+) in the tumor microenvironment. In addition, our results revealed that *L. acidophilus* lysates had an immunomodulatory effect through inhibition the M2 polarization and the IL-10 expressed levels of LPS-activated Raw264.7 macrophages. Finally, the 16S rRNA gene sequencing of fecal microbiota demonstrated that the combined administration significantly inhibited the abnormal increase in the relative abundance of proteobacteria and partly counterbalance CRC-induced dysbiosis in model mice. Overall, these data support promising clinical possibilities of *L. acidophilus* lysates with CTLA-4 mAb in cancer patients and the hypothesis that probiotics help shape the anticancer immune response.

## Introduction

Colorectal cancer (CRC) is the fourth leading cause of cancer-related death worldwide. The development of this disease may be attributed to numerous factors such as gene mutations, family history, dietary composition, and inflammatory dysregulation^1,2^. Recently, valuable insights have been gained into the role of the intestinal microbiota in CRC development, and extensive investigations have suggested that intestinal microbial homeostasis is closely related with above pathogenic factors of CRC^3–5^.

After birth, the microbiota of an individual is mainly shaped by maternal factors related to birthing and postnatal factors such as diet and environment^6,7^. These microbiota offer nutritional, immune defense, and immune development-related development benefits to the host^8,9^. Microorganisms colonizing in the intestine are in a dynamic equilibrium^10^, and the composition of the microbial community may be affected by alcohol consumption, diet, and the intestinal micro-environmental factors such as pH, mucosal integrity, and nutrient availability^11^. Additionally, growing evidence shows that abnormal gut microbiota participate in various pathophysiological processes associated with many diseases, including CRC^4,5^. However, the specific roles of each microbe in CRC development are unclear.

Previous studies have shown that some probiotic bacteria, such as *Bifidobacterium longum*^12^*, Lactobacillus acidophilus*^13^, and *Enterococcus faecalis*^14^, mediate inflammatory responses, inhibit the proliferation of malignant cells, and reduce levels of pro-carcinogenic metabolites in CRC. Additionally, Aghazadeh *et al*. reported that the secretions of an *Acetobacter syzygii* strain exerted anti-cancer effects against squamous cell carcinoma^15^.

Preliminary data have shown that probiotic lysates, unlike live microbes, may be administered therapeutically without potential adverse side effects, which are particularly oriented towards immune system regulation^16,17^. Furthermore, the use of probiotic lysates would allow the manufacturers of probiotic products to circumvent the logistical challenges of maintaining the viability of bacteria during formulation, transportation, and preservation^18^. The objective of the present study was to determine whether a combination of administered probiotic lysates and checkpoint blockade could enhance antitumor immune responses. We evaluated the efficacy of the lysate of *L. acidophilus* and anti-mouse (m) CTLA-4 antibodies, administrated alone or in combination, in mouse CRC models chemically induced using azoxymethane (AOM) and dextran sodium sulfate (DSS).

## Materials and Methods

### Preparation of cell lysates

**Figure 1:**
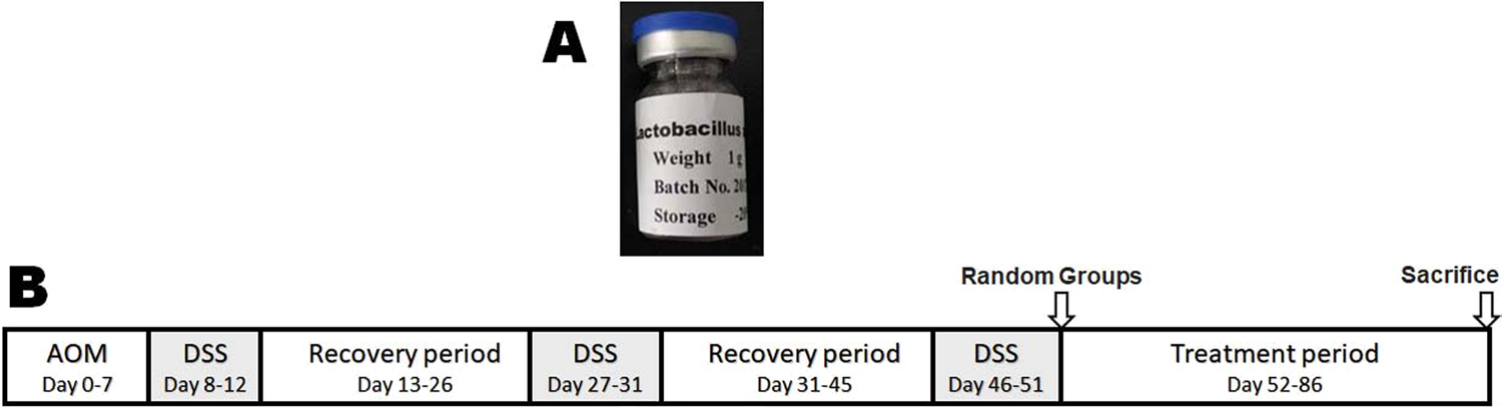
Schematic time schedule of combination therapy with anti–CTLA-4 antibodies and probiotic lysates (**A**) Lysates of *Lactobacillus acidophilus*. (**B**) Experimental protocol for the induction of colorectal cancer and combination therapy in BALB/c mice. A single intraperitoneal injection of 10 mg/kg azoxymethane (AOM) at 7 days was followed by three cycles of administration of 2% dextran sulfate sodium (DSS) in sterile drinking water for 5 days, followed by normal drinking water for another 14 days. Then, the following two experiments were performed. Experiment 1# (Lysates therapy every other day from day 52), Low-dose group (i.g., lysates of 5 × 10^6^ CFU *L. acidophilus*/mouse); High-dose group (i.g., lysates of 2 × 10^7^ CFU *L. acidophilus*/mouse) and PBS control group (i.g., equivalent volume PBS). Experiment 2# (Combined therapy every other day from day 52), CTLA-4 IgG group (50 μg/mouse); Lysates group (i.g., lysates of 5 × 10^6^ CFU *L. acidophilus*/mouse); Combined group (50 μg/mouse i.p. and the equivalent lysates i.g.); Model control group (equivalent volume PBS, i.p. and i.g.) and Normal control group (equivalent volume of PBS, i.p. and i.g.).

*L. acidophilus* (ATCC 33198) were purchased from China General Microbiological Culture Collection Center (CGMCC), Beijing, China, and were cultured in 10 L MRS broth at 37 °C. After 24 h, the cells were harvested by centrifuging at 4,000 × g (4 °C and 20 min), washed with phosphate-buffered saline (PBS) three times. Then, the cell pellet was re-suspended in PBS at a concentration of 10^8^ CFU/ml, and disrupted at 1,200 bar (4 °C, 2 min/time, 3 times) with JN-6000C PLUS low-temperature ultrahigh-pressure continuous flow cell crusher (JNBIO, Guangzhou, China)^19^. Finally, after centrifuged at 4,000 × g (4 °C and 20 min) to remove any whole bacteria remaining, the lysate of *L. acidophilus* was freeze-dried in a bottle (Fig. 1A). Before intragastric administration (i.g.), the freeze-dried lysates were dissolved in ddH_2_O at a concentration of 10^8^ CFU/ml.

### Animals and experimental design

All experiments were performed in accordance with institutional ethical guidelines, and the study was approved by the Ethics Committee for Animal Research of Nanjing Medical University. Healthy male BALB/c mice (6–8 weeks old) were obtained from the Model Animal Research Center of Nanjing University (Nanjing, China) and housed in specific pathogen-free conditions at Nanjing First Hospital Animal Center, Nanjing, China, in a room controlled for temperature (21 ± 2 °C), humidity (55 ± 5%), and light (12-h light/dark cycle).

For generating colitis-associated CRC models, mice (with the exception of normal negative control) were induced with a single intraperitoneal injection of 10 mg/kg azoxymethane (AOM) (Sigma-Aldrich, St. Louis, MO) 7 days, and subsequently induced by three cycles of 2% dextran sulfate sodium (DSS) (MP Biomedicals, Santa Ana, CA) in sterile drinking water for 5 days, followed by normal drinking water for another 14 days (Fig. 1B), as previously described^20^. On day 52, the induced mice were randomly assigned to different groups (n = 10/group) as following.

1. Mice were administrated by lysates every other day for 5 weeks as follows: low-dose group (i.g., lysates of 5 × 10^6^ CFU *L. acidophilus/mouse*), high-dose group (i.g., lysates of 2 × 10^7^ CFU *L. acidophilus/mouse*) and PBS control (i.g., equivalent volume PBS).
2. Mice then received combined therapy every other day for 5 weeks as follows: CTLA-4 IgG group (anti-mouse-CTLA-4 IgG in PBS, clone# 9H10, intraperitoneal injection i.p., 50 μg/mouse)^21^; lysates group (i.g., lysates of 5 × 10^6^ CFU *L. acidophilus/mouse*); combined group, (equivalent dose anti-CTLA-4 IgG i.p. and lysates i.g.) and model control group (equivalent volume PBS, i.p. and i.g.). The mice in the normal control group were also treated with an equivalent volume of PBS, i.p. and i.g.

### *In vivo* safety evaluation of *L. acidophilus* lysate in mice

A 4-week study was conducted in male BALB/c mice to evaluate the toxicity of administered lysate of *L. acidophilus*. The study-specific protocol was approved by the Ethics Committee for Animal Research of Nanjing Medical University. Thirty mice were randomized into 3 groups (n = 6/group): Low does lysates (i.g., lysates of 5 × 10^6^ CFU *L. acidophilus*), High does lysates (i.g., lysates of 2 × 10^7^ CFU/ml. acidophilus), or PBS control group (i.g., 200 μl/mouse PBS). After receiving an intragastric administration once a day for 28 days, the mice were evaluated for changes in clinical signs daily and body weights were recorded weekly. After the last lysate dose, all mice were euthanized by CO_2_ inhalation on day 29. Colorectal tissues were processed for microscopic and histologic evaluations, and lymphocyte subsets in peripheral blood and mesenteric lymph nodes were analysed by flow cytometry on day 29.

### Morphometric measurements and symptom severity

Body weights were monitored weekly over the duration of the experiment. Symptom severity was assessed twice/week beginning at the first cycle of DSS exposure, by symptom score as previously described^22^, which included body weight loss (WL) (0 = < 5%, 1 = 6–10%, 2 = 11–15%, 3 = > 15%); stool consistency (0 = pellet, 2 = pasty, and 4 = diarrhea); occult/gross rectal bleeding assessed by the urine fecal occult blood test kit (Shanghai Yisheng Biological Technology Co., Ltd.) (0 = negative, 2 = positive, and 4 = gross bleeding). Mice were sacrificed at day 85. Colons tissues were collected, cleaned with PBS, and stored under required conditions for subsequent analyses.

### Histology and immunohistochemistry of the colon

Whole-colon tissues were thoroughly rinsed and then fixed in 10% formalin for 24 hours. After each group of colon tissues was embedded together with a paraffin block, the fixed tissues were sectioned and stained by hematoxylin/eosin (H&E) staining and immunohistochemical staining with primary rabbit anti-mouse CD8a (Cell Signaling Technology, #98941, 1:400), rabbit anti-mouse Foxp3 (Cell Signaling Technology, #12653, 1:100), or rabbit anti-mouse Granzyme B (Cell Signaling Technology, #46890, 1:125), and then anti- rabbit IgG-HRP secondary antibodies. Finally, a pathologist who was blinded to the clinical data for all mice evaluated all sections for numbers of tumors and immunohistochemical staining of whole-colon tissues.

### Reverse transcription and real-time quantitative PCR analysis

The cytokine mRNA levels in peritoneal macrophages and mesenteric lymph nodes were analyzed by quantitative real-time PCR (qRT-PCR), as previously described. Briefly, primary peritoneal macrophages were isolated from the sacrificed mice at day 85 by injecting their peritoneal cavity with 10 ml of Dulbecco’s modified Eagle’s medium (DMEM), massaging, and then drawing back to the syringe the fluid now containing the macrophages. After centrifugation, cells were resuspended in DMEM with 10% fetal bovine serum (FBS) (Gibco, Life Technologies, USA) and 1% v/v penicillin and streptomycin (Gibco, Life Technologies, USA), and were plated 6-well plates for 2 h to facilitate adherence at 37 °C in a 5% CO_2_ incubator. The adherent cells, regarded as primary peritoneal macrophages, were analyzed the cytokine mRNA levels by qRT-PCR.

The qRT-PCR was performed in both the peritoneal macrophages and mesenteric lymph node tissue following RNA isolation with TRIzol reagent (Invitrogen Life Technologies, Paisley, UK). The cDNA template was synthesized using a HiScript^®^ 1^st^ Strand cDNA Synthesis kit (Vazyme Biotech Co., Ltd, Nanjing, China); then, qPCR analysis was performed using a AceQ Universal SYBR Green qPCR Master Mix (Vazyme Biotech Co., Ltd, Nanjing, China), according to standard protocols, using TNF-α, IL-2, IL-10, and IFN-γ primers. Relative gene expression was normalized to the expression of actin. All primer sequences are shown in Table S4.

### Cell preparation and flow cytometric analysis

We assessed lymphocyte subsets from the mesenteric lymph node samples by flow cytometry. Briefly, single cell suspensions were prepared by smashing Peyer’s patches and mesenteric lymph node tissues with a syringe and nylon mesh, and washing with PBS. Then, we stained the cells (100 μL) with the different antibody panels at working concentrations as follows: FITC Rat Anti-Mouse CD4 (Clone# H129.19), PE-Cy™5 Rat Anti-Mouse CD8a (Clone# 53–6.7), APC anti-Mouse Foxp3 (Clone# 3G3), PE Rat Anti-Mouse CD25 (Clone# PC61), FITC Rat Anti-Mouse CD44 (Clone# IM7), PE-Cyanine7 anti-mouse IFN-γ (Clone# XMG1.2) and PE-Cyanine7 anti-mouse CD62L (Clone#MEL-14). Additionally, peritoneal macrophages were collected from mice injected with 5 mL of PBS and stained with the panel of PerCP-Cyanine5.5 anti- mouse F4/80 (Clone# BM8), PE anti-mouse CD206 (Clone#MR6F3) and FITC anti-mouse CD11b (Clone#M1/70). After incubation in the dark at 4 °C for 20 min and washing twice in PBS, the stained cells were analyzed using a FACSCanto™ II flow cytometer (BD Biosciences, San Diego, CA, USA). Finally, the data were analyzed using FlowJo software (Treestar, San Carlos, CA, USA).

### Isolation of bacterial genomic DNA and quantitative PCR determination of microbiota 16S ribosomal RNA gene sequencing

After aseptic collection from each mouse, the feces were kept in sterile tubes and immediately frozen at −80 °C for microbial DNA extraction and analysis of bacterial community composition. Briefly, PCR products were amplified from the V4-V5 regions of the bacterial 16S ribosomal RNA gene using primers of 27 F (AGAGTTTGATCCTGGCTCAG) and 533 R (TTACCGCGGCTGCTGGCAC), purified using the AxyPrep DNA Gel Extraction Kit (Axygen Biosciences, Union City, CA, USA from 2% agarose gels, and then paired-end sequenced on an Illumina MiSeq platform (Majorbio Biomedical Technology Co., Ltd, Shanghai, China) according to standard protocols. Finally, all sequence data were analyzed by the Ribosomal Database Project (RDP) Classifier against the Silva 16S rRNA database (SSU117) with a confidence threshold of 70%^23^.

### Evaluation of effects of *L. acidophilus* lysates on macrophages *in vitro*

The macrophage RAW 264.7 cell line (purchased from Shanghai Cell Bank, Chinese Academy of Sciences) were cultured in complete Dulbecco’s modified Eagle’s medium (DMEM) (Gibco, Carlsbad, CA) with 10% fetal bovine serum (FBS) (Gibco) and 1% v/v penicillin and streptomycin (Gibco) at 37 °C in a 5% CO_2_ incubator. Briefly, the cells were cultivated in the presence of different protein concentrations of bacterial lysate (filtered by the 0.22 micron membrane), or sterile PBS in the presence or absence of 1 μg/ml lipopolysaccharide (LPS, Sigma-Aldrich). After cultivation 24 h, we analyzed the mRNA levels of tumour necrosis factor α (TNF-α) and IL-10 by qRT-PCR and the CD206 (macrophage mannose receptor) expressed levels of RAW264.7 cells by flow cytometric analysis.

### Statistical analysis

Data were analyzed with GraphPad Prism version 5 (San Diego, CA, USA). Two-tailed Student’s *t*-tests were used to compare experimental groups. Statistical significance was defined at greater than or equal to a 95% confidence interval or a *p*-value < 0.05. The data shown are the mean ± standard deviations of 3 repeated experiments.

## Results

### *L. acidophilus* lysate inhibited tumour development in AOM/DSS-treated mice

**Figure 2:**
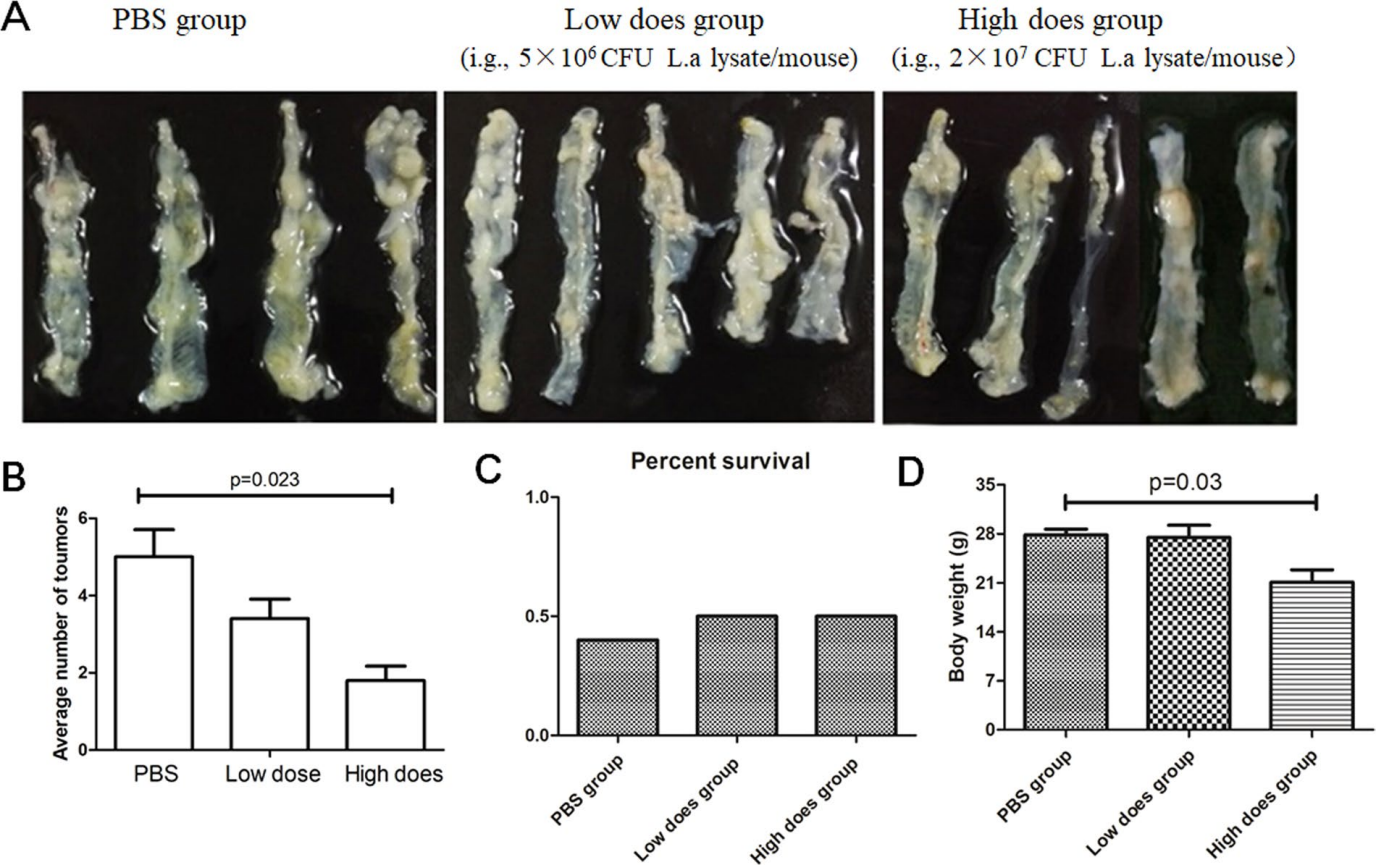
*L. acidophilus* lysates inhibited tumor formation in AOM/DSS model mice. (**A**) Views of tumors in the colorectal of AOM/DSS model mice after 86 days. Colic tumor number in the colons (**B**), Survival rate (**C**) and Body weight of AOM/DSS mice treated PBS, low-dose lysates and high-dose lysates (i.g.) after 86 days. Data are represented as the mean of each group ± SD.

**Figure 3:**
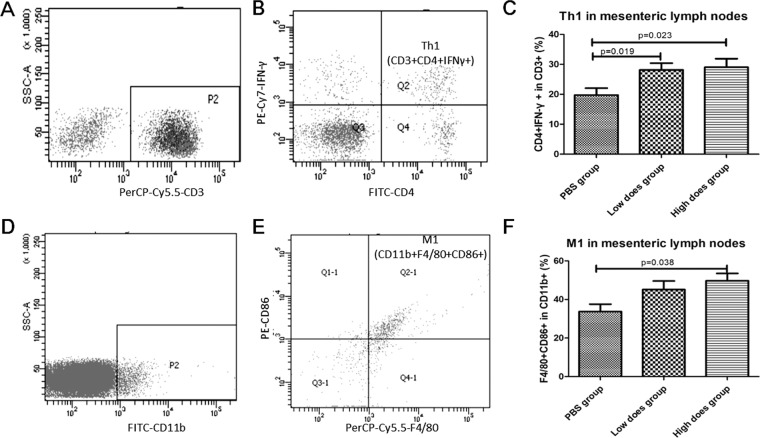
*L. acidophilus* lysates increased the proportion of Th1 cells and M1 macrophages in the mesenteric lymph nodes. After the 4-week safety evaluation study of *L. acidophilus* lysates (i.g., Low does lysates, lysates of 5 × 10^6^ CFU *L. acidophilus*/mouse; High does lysates, lysates of 2 × 10^7^ CFU *L. acidophilus*/mouse), the cells in mesenteric lymph nodes (mLNs) of each mouse were isolated, respectively stained with a panel of CD3 + CD4 + IFN-γ + (for Th1 cells, **A**,**B**) CD11b + F4/80 + CD86 + (for M1 macrophages, **D**,**E**), and assessed by flow cytometry. (**C**,**F**) The right column charts represent the statistical data shown in the left plot. Control group, 200 μl PBS/mouse (i.g.). Data (n = 10/group) are represented as the mean of each group ± SD.

Previous paper reported that lysate of *L. actobacillus* could ameliorate colitis by strengthening the gut barrier function and changing the gut microenvironment in DSS-induced colitis model mice^24^. As shown in Fig. 2, as compared with PBS or low-dose lysates, administration of high-dose lysates could significantly reduce the number of visible tumors and average body weight in the colitis-associated CRC models. Therefore, we conducted a safety study in healthy mice to assess the potential toxicity of L. acidophilus lysates. Compared to the PBS control group, although no difference in T cell subsets (CD3 + CD4 + and CD3 + CD8 + ), Treg (CD4 + CD25 + Foxp3 + ), B lymphocytes (CD19+), NK cells (DX5 + ) in mice PBMCs and mesenteric lymph nodes were observed among all groups on day 29 (Supplementary Table S1 and S2), there was a significant enhancement in lymphocyte subsets detected for Th1 helper lymphocytes (CD3 + CD4 + IFN-γ+) and M1 macrophages (CD11b + F4/80 + CD86+) in mesenteric lymph nodes in the Low does group and High does group (Fig. 3, *p* < 0.05), indicating that L. acidophilus lysates have the effects immunological adjuvant to activate the immune response. Histological examination showed, compared to the PBS control and low-does lysates (lysates of 5 × 10^6^ CFU *L. acidophilus*), high-dose lysates (lysates of 2 × 10^7^ CFU *L. acidophilus*) enhanced infiltration of inflammatory cells in gut mucosa, representing a local immune response caused by the gastrointestinal administration of excessive and long-termed lysates (Supplementary Figure S1). In addition, throughout the course of the study, no significant differences were observed in body weight, food consumption, and stool consistency daily (Supplementary Table S3).

### *L. acidophilus* lysates pre-treatment inhibited the production of IL-10 in LPS-activated macrophages *in vitro*

**Figure 4:**
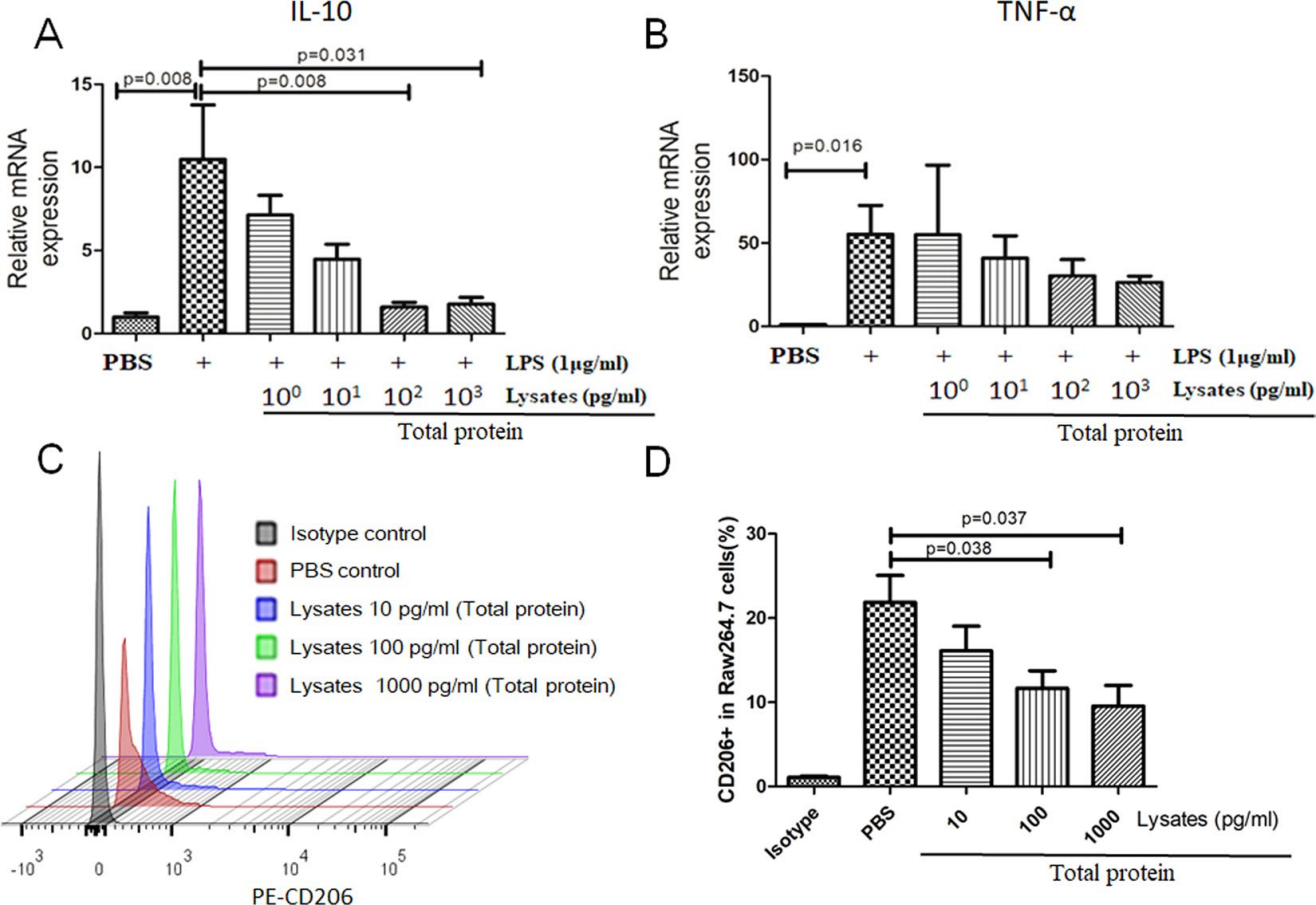
*L. acidophilus* lysates inhibited the CD206 expression of RAW 264.7 macrophages and the IL-10 mRNA levels of LPS-activated RAW 264.7 macrophages. (**A**) *L. acidophilus* lysates could decrease the mRNA expressed levels of IL-10 in 1 mg/l LPS-activated macrophages, while could not decrease the TNF-α levels (**B**). The results of flow cytometry suggested that L. acidophilus lysates mediated M2 polarization by inhibition the CD206 receptor on RAW 264.7 macrophages (**C**). (**D**) The right column charts represent the statistical data shown in the left plot. The lysate was quantified by the total protein concentration. Data are represented as the mean of each group ± SD.

Because probiotics have an immunomodulatory effect on the innate immunity systems, we analysed the anti-inflammatory effect of *L. acidophilus* lysates on LPS-activated RAW 264.7 macrophages *in vitro*. As shown in Figs. 4A, 1 μg/ml LPS significantly increased the mRNA and protein levels of IL-10 in the RAW 264.7 cells *in vitro*, which could be inhibited by the *L. acidophilus* lysates. In addition, the treatment with the lysates did not change the TNF-α production in LPS treated macrophages. As published by others^25^, this result suggests that the lysates could make a difference to the polarization stage of macrophages leaded by LPS. The results of FCM showed that the CD206 receptor (M2 phenotype marker) was significantly downregulated in lysates treated macrophages (Fig. 4C,D). Therefore, *L. acidophilus* lysates seems to inhibit M2 polarization and reduce the production of IL-10 in macrophages under inflammatory conditions.

### Effects of combined administration of lysates and anti-CTLA-4 IgG on morphometric characteristics in AOM/DSS-treated mice

**Figure 5:**
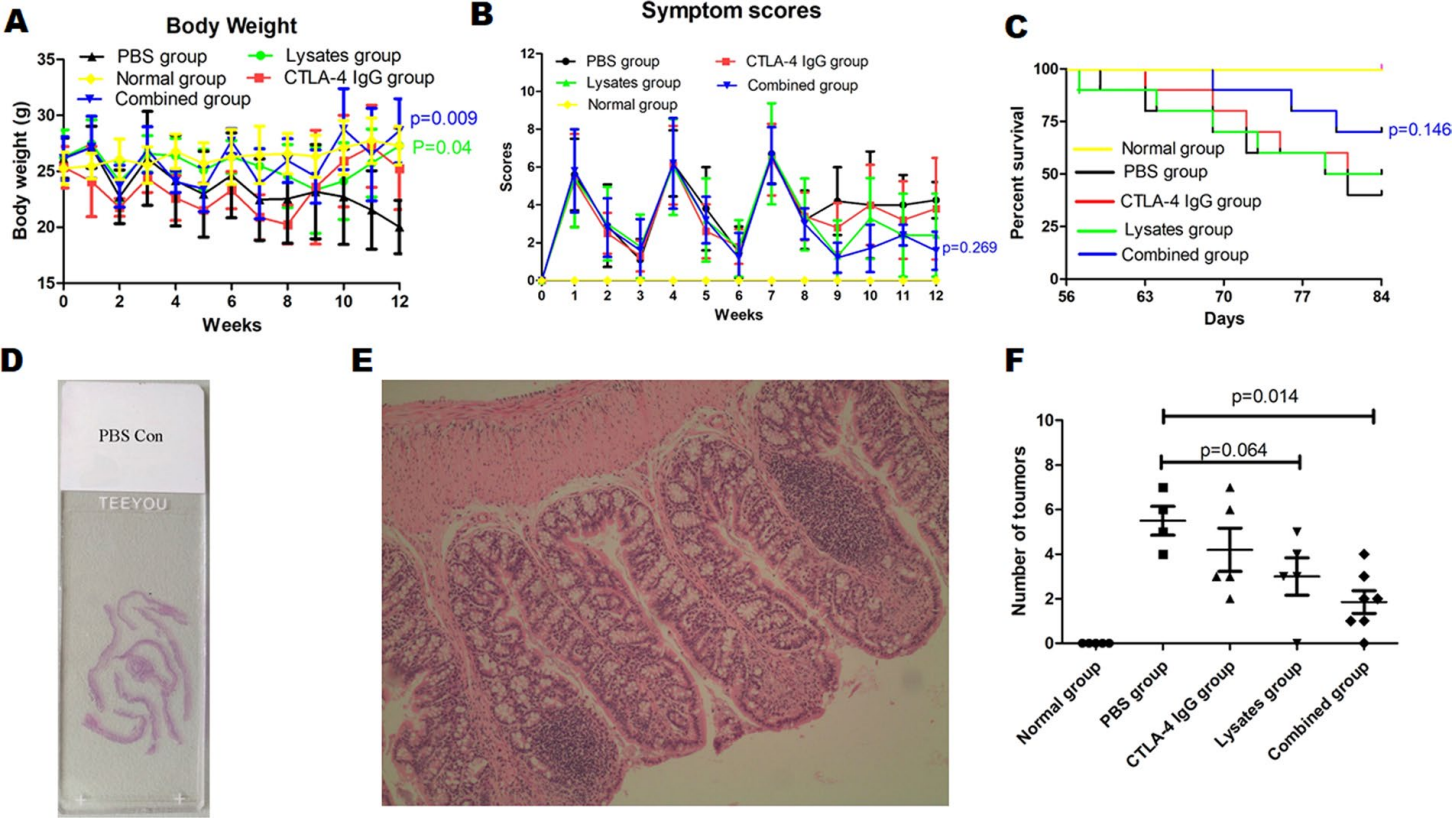
Combination therapy with anti–CTLA-4 and probiotic lysates protected against tumor formation. (**A**) Mouse body weight measurements through the duration of the study. Body weight in the combined group and in the lysate group was significantly higher than that in the PBS control group (*p* = 0.009 and 0.04 *vs*. PBS group). (**B**) Symptom score (body weight loss + blood in stool + diarrhea) through the duration of the study (*p* = 0.269, combined group *vs*. PBS group). (**C**) After randomly dividing into 4 treatment groups (n = 10/group) at day 51, the AOM/DSS-induced mice were treated with different therapies for 5 weeks, and survival was recorded accordingly (*p* = 0.146, combined group, Survival rate 70% *vs*. PBS group, Survival rate 40%). (**D**–**F**) Histological assessment of tumor incidence in the whole colorectal of mice model. Each group of colon tissues was embedded together with a paraffin block, representative slide with all colorectal tissues in the PBS group (**D**). (**E**) Representative H&E-stained images of colic tissues from mice at sacrifice in PBS group. Images were acquired using the Olympus BX50 microscope (magnification, ×100). (**F**) Colic tumor number in the whole colons of treated mice. Data are represented as the mean of each group ± SD.

Then, to determine the potential beneficial effects of lysates on immunotherapy in colorectal cancer, combined administration of lysates and anti-CTLA-4 IgG was started at day 51 and performed once every two days for the duration of this study. As shown in Fig. 5, there was no difference in average body weight and symptom scores among therapy groups before combined administration; however, the administration of lysates alone or in combination with anti-CTLA-4 IgG significantly attenuated the loss of body weight (Fig. 5A, *p* < 0.05 or *p* < 0.01). Although combined administration partially decreased symptom scores and increased the survival rates of model mice, there were no statistical difference between the combined-treatment group and PBS control group (*p* > 0.05) (Fig. 5B,C).

The whole-colon tissues in different groups were observed with microscope after H&E staining, and the results showed each mouse in PBS control group developed 4–7 tumors in the colorectal. Although alone administration of anti-CTLA-4 IgG or lysates could not significantly reduce the incidence of tumors (*p* > 0.05), mice treated by combined administration developed fewer and smaller tumors in whole colorectal than the PBS control mice (*p* = 0.014) (Fig. 5D–F). Although some animals have some improvement in body weight, symptom scores, percentage of survival and numbers of tumors in the lysates group or anti-CTLA-4 IgG group, but there was no statistical difference compared to the PBS control over the course of the experiment (*p* > 0.05).

### Lysates enhanced anti-tumor responses generated by anti-CTLA-4 IgG in tumor-draining mesenteric lymph node

**Figure 6:**
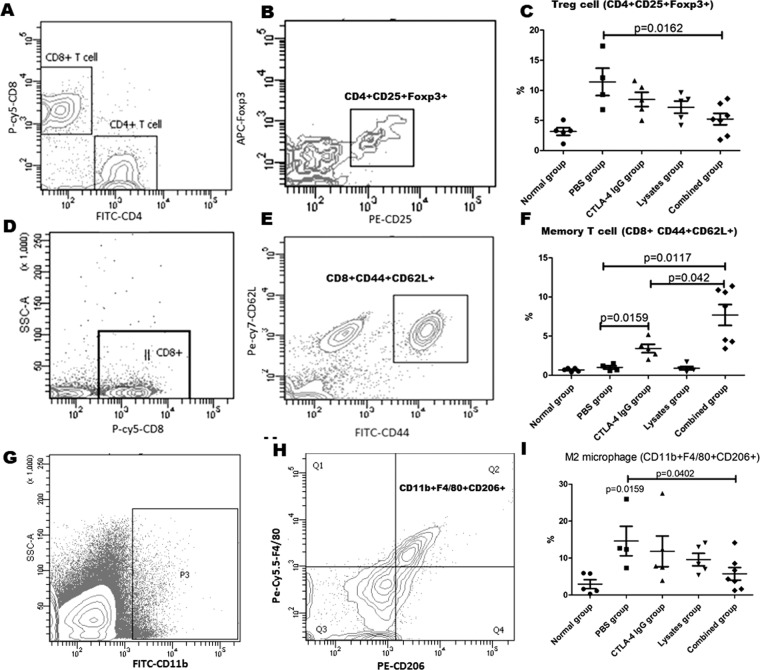
Combination therapy with anti–CTLA-4 and probiotic lysates enhances anti-tumor responses in tumor-draining mesenteric lymph nodes of the CRC model mice. After therapy, the cells in mesenteric lymph nodes (mLNs) of each mouse were isolated, respectively stained with a panel of CD4/CD8/CD25/Foxp3 (for Treg cells, **A** and **B**) CD8/CD44/CD62L (for memory CD8 + T cells, D and E), or CD11b/F4/80/CD206 (M2 macrophage cells, G and H), and assessed by flow cytometry. (**C**, **F**, and **I**) The right scatter charts represent the statistical data shown in the left plot. Data are represented as the mean of each group ± SD.

We next investigated the effect of the combinatorial treatments on lymphocyte proportions in tumor-draining mesenteric lymph nodes (mLNs). CD4 + CD25 + Foxp3 + Treg cells and CD11b + F4/80 + CD206 + M2 macrophages in mLNs of CRC models were significantly increased compared with those in normal mice (*p* < 0.05). When lysates were administered in combination with anti-CTLA-4 mAb in this study, not only were the abnormal changes in Treg and M2 cells in CRC models suppressed, but memory CD8 + T cell (CD8 + CD44 + CD62L + ) numbers were increased compared with that in response to single anti-CTLA-4 mAb agent treatment (*p* < 0.05) These findings suggest that combinatorial treatments may increase the accumulation of anti-tumor responses in tumor-draining mesenteric lymph nodes (Fig. 6).

**Figure 7:**
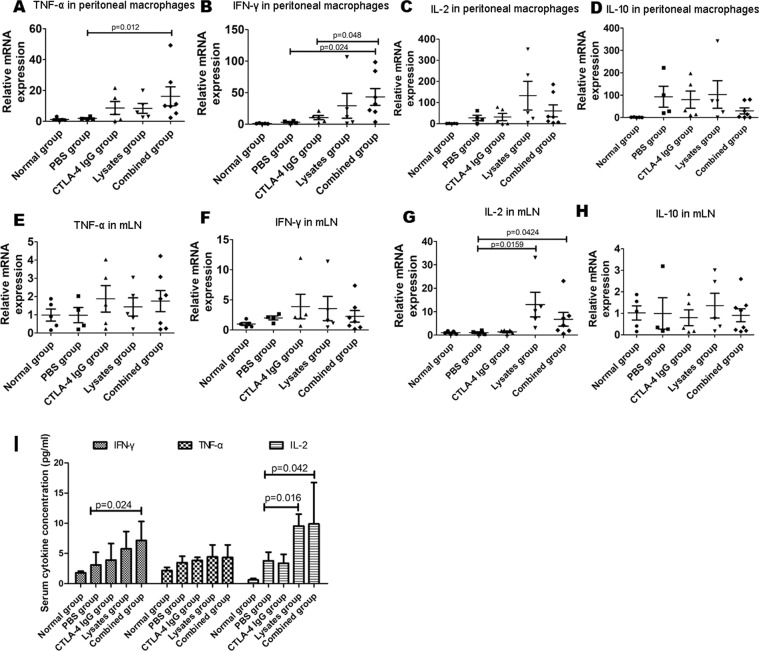
Analysis of tumor-immunity-associated cytokine expression by RT-PCR and ELISA. Total RNA was extracted from peritoneal macrophages (**A**–**D**) and mLNs (**E**–**H**) of each group to quantify the mRNA expression levels of IFN-γ, TNF-α, IL-2, and IL-10. (**I**) ELISA analysis of IFN-γ, TNF-α, and IL-2 in serum. Data are represented as the means of each group ± SD of three independent experiments.

To verify this hypothesis, we investigated the mRNA expression levels of TNF-α, IL-2, IFN-γ, and IL-10 in the peritoneal macrophages (Fig. 7A–D) and mLNs (Fig. 6E–H). Compared with the PBS control, combinatorial treatments significantly enhanced the mRNA expression levels of TNF-α and IFN-γ in the peritoneal macrophages and the IL-2 mRNA levels in mLNs (*p* < 0.05); further, the IFN-γ mRNA levels in the peritoneal macrophages were significantly higher in the combined group than in the anti-CTLA-4 IgG group (*p* = 0.048). Additionally, as shown in Fig. 6I, relative to the PBS control, combinatorial treatments significantly enhanced the IFN-γ and IL-2 levels in serum (*p* < 0.05).

### Lysates promoted infiltration of T cells in the tumor microenvironment after combined treatment with CTLA-4 blocking antibody in AOM/DSS-treated mice

**Figure 8:**
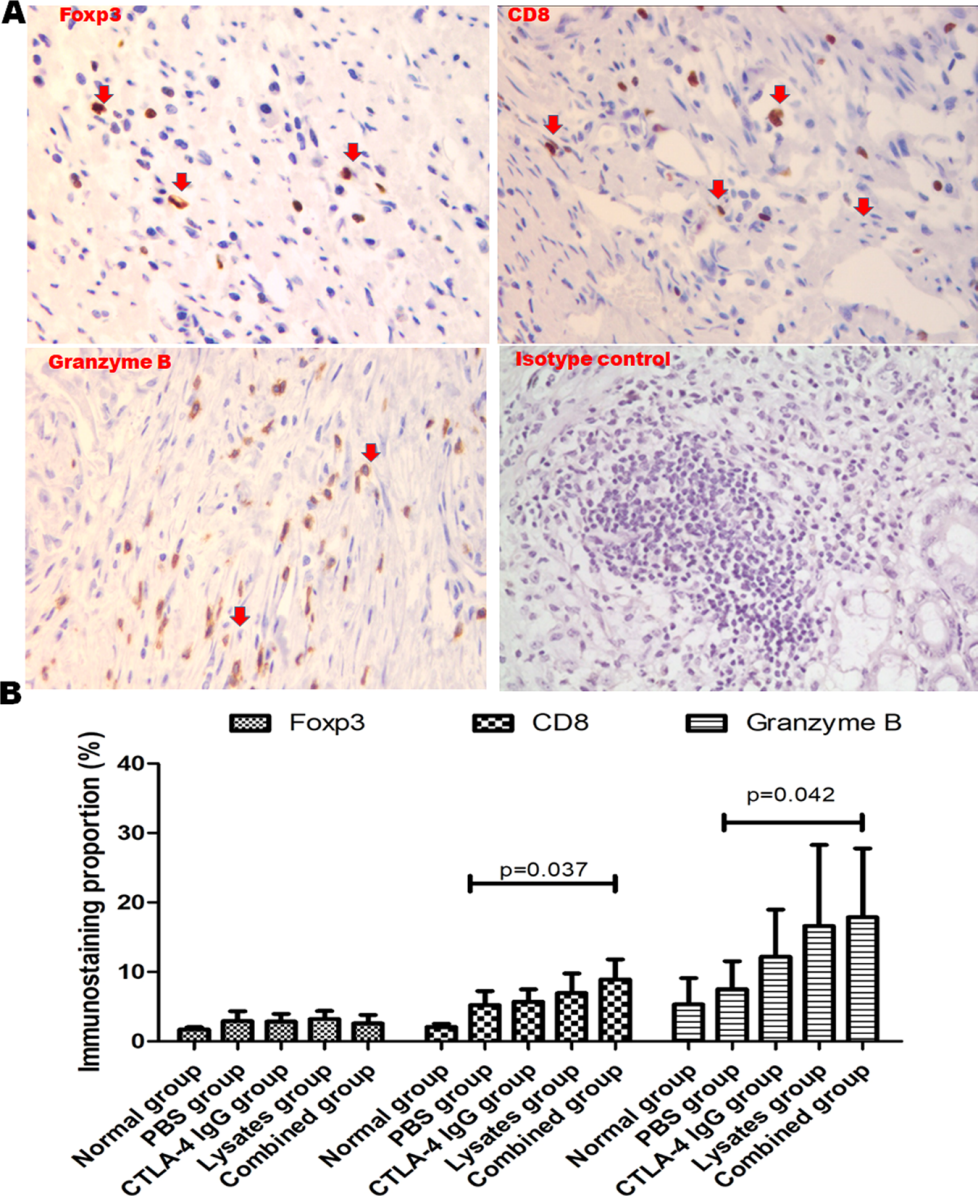
Combination therapy with anti–CTLA-4 and probiotic lysates enhances the infiltration of CD8+ T cells in the tumor microenvironment of CRC model mice. (**A**) Representative immunostaining images for Foxp3, CD8, and Granzyme B of colic tissues from mice at sacrifice. Images were acquired using the Olympus BX50 microscope (magnification, ×400). (**B**) Quantification of immunostaining was conducted using ImageScope software to calculate the proportion of positive nuclear area occupied by Foxp3, CD8, and Granzyme B, divided by total nuclear area based on completion of scanning of the entire section. Data are represented as the mean of each group ± SD.

To explore the immune response against tumors in CRC model mice, we performed immunohistochemical analysis (IHC) to analyze the Foxp3, CD8, and Granzyme B (Fig. 8A) expression after therapy; this was quantified as the number of positive cells divided by total cells in the tumor microenvironment. Representative images of whole colorectal sections with immunohistochemical staining are shown in Fig. 8. Quantitative analysis of the immunostained sections indicated that the proportion of cells staining positive for CD8+ and Granzyme B (cytolytic cytokines against tumor) in the tumor microenvironment was significantly higher after combined treatment with lysates and anti-CTLA-4 IgG than after treatment with the PBS control (Fig. 8B); however, no statistical difference was observed among the lysate group, anti-CTLA-4 IgG group, and the combined group (*p* > 0.05). Additionally, there was no difference in the percentage of Foxp3 + −positive staining in the tumor microenvironment among the lysate group, anti-CTLA-4 IgG group, the combined group, and PBS control group (*p* > 0.05).

### Lysates ameliorated the dysregulation of microbiota in the gut of AOM/DSS-treated mice

**Figure 9:**
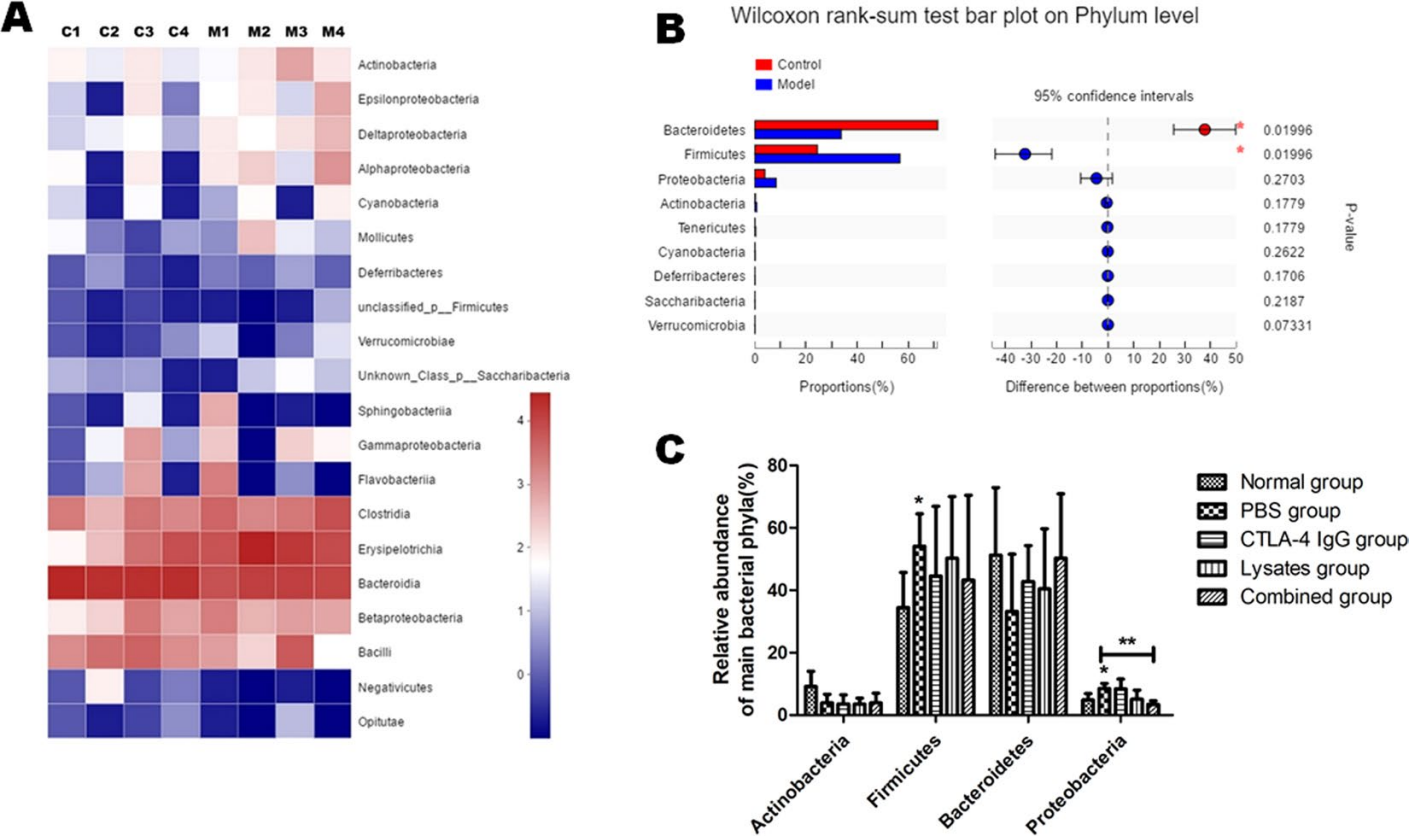
Combination therapy with anti–CTLA-4 and probiotic lysates ameliorated the changes in microbial composition in feces form CRC mice models induced by AOM/DSS. (**A** and **B**). Microbial composition in feces from mice before AOM/DSS controls (Control, C1-C4) and after AOM/DSS treatment (Model, day 50, M1-M4) was analyzed by 16S ribosomal RNA gene sequencing. (**A**) Heatmap coloring indicates the relative abundance of the OTU found in AOM/DSS mice feces. (**B**) The significance of differences in diversity of flora between AOM/DSS-treated mice and before-AOM/DSS-treatment controls was tested using the Wilcoxon rank-sum test bar plot on phylum levels; *p < 0.05 *vs*. control. (**C**) Sequencing analysis and 16S ribosomal RNA PCR to determine the proportion of *Bacteriodetes, Firmicutes, Proteobacteria*, and *Actinobacteria* in the total bacterial load of fecal samples from mice after therapy; *p < 0.05 *vs*. Normal group; **p < 0.01. Data are represented as the mean of each group ± SD.

Microbiota homeostasis is critical for maintaining intestinal physiological function. Dysregulation of microbiota has been shown to contribute to CRC pathogenesis. In order to identify the microbiota related with worsened CRC pathology in the AOM-DSS models, we next performed 16S rRNA gene sequencing to explore the differences in bacterial diversity among fecal samples from individual mice before AOM injection (Control) and one day before therapy (Model). As shown in Fig. 9A,B, in comparison with that observed in normal mice, the fecal microbial community in model mice was altered in response to AOM/DSS treatment: in particular, the proportion of *Bacteroides* was significantly decreased and that of *Firmicutes* was significantly increased (*p* < 0.05). Quantitative PCR was performed to determine if the lysates of *L. acidophilus*, which belong to *Firmicutes*, affected the microbial community in mouse feces after therapy. Based on 16S primers specific for phylum, a total of 4 bacterial phyla (more than 1%) were predominantly detected in the feces of the experimental mice. The relative abundance of bacteria from the phyla *Proteobacteria* and *Firmicutes* was significantly higher in the PBS group than in the normal control group (*p* < 0.05). Additionally, the combined administration of CTLA-4 IgG with lysates significantly inhibited the abnormal increase in the relative abundance of *Proteobacteria* compared with that in the other therapy groups (*p* < 0.01) (Fig. 9C).

## Discussion

Therapeutic strategies utilizing immune checkpoint inhibitors (such as anti-CTLA4 and anti-PD-1 antibodies) have been used to induce anti-tumor immunity in T cells, achieving promising clinical benefits in many types of cancer^26^. However, such immunotherapy is still in its infancy partly due to the specific tumor microenvironment of CRC^27,28^. CRC, which develops in the colon or rectum, is considered a bacterial-related disease in humans^3,29^. A growing body of data show that gut microbes may shape response to cancer immunotherapy^30^, and that intratumoral injection of attenuated bacteria inhibits tumor growth in several animal models^31^. In this study, we showed that lysates of *L. acidophilus* combined with anti-CTLA-4 antibody blockade enhanced antitumor immunity in mouse CRC models induced by AOM/DSS, by synergistically improving anti-tumor T cell immunity.

Previous investigations have shown that probiotic bacteria, including *B. bulgaricus, B. subtilis*, and *L. acidophilus*, not only alter the intestinal microflora of colon cancer patients, but also secrete effector molecules to inhibit tumor initiation and progression in animal tumor models^32,33^. Additionally, Zakostelska *et al*. reported that oral treatment with lysate of *L. casei DN-114 001* ameliorated acute DSS-induced colitis in BALB/c mice by changing the gut microbiota composition and modulating the mucosal immune system, as evidenced by an increase in Treg cells in mLN, and a decrease in TNF-a, IFN-γ, and IL-10 levels in Peyer’s patches^25^. However, in this study, we demonstrated that the lysates alone partially reversed the dysregulation of homeostasis in the fecal microbial community induced by AOM/DSS treatment in BALB/c mice, significantly increased the CD8 + CD62L+ T cells in mLN of CRC mice, and did not affect TNF-a and IL-10 expression levels. Differences in probiotic efficacy reported by various studies indicate that probiotic function may be affected by numerous factors, including production parameters (fermentation conditions, concentration techniques), product parameters (strain, product composition), and host parameters (host microbiota, immune factors). In addition, it is well known that probiotics exert strain-specific effects. When selecting a strain, four *Lactobacillus acidophilus* (ATCC 11975, 33200, 11506, 19992 and 33198) were compared for their ability to produce lactic acid, and ATCC 33198 is the strain that produces the highest lactic acid. Frankly, we should compare their ability of enhance antitumor immunity *in vivo*, and screen the most suitable strains to study its drug properties.

Numerous clinical trials have shown that ipilimumab, a fully human monoclonal antibody (Ab) directed against CTLA-4, has been successfully used as immunotherapy to treat many different types of cancers, including melanoma, renal cell carcinoma, prostate cancers, urothelial carcinoma, and ovarian cancer, and that the therapeutic effect correlates with selective depletion of Treg cells in the tumor microenvironment^34,35^. However, owing to its poor efficacy and severe adverse events in the intestinal tract (diarrhea, abdominal pain, rectal bleeding, and nausea), ipilimumab is rarely used for treatment for colorectal cancer. Additionally, Vétizou *et al*. found that the antitumor effects of CTLA-4 blockade depend on distinct *Bacteroides* species^36^. In this study, the results of a 28-day safety study suggested that high-dose lysates (lysates of 2 × 10^7^ CFU *L. acidophilus*) enhanced infiltration of inflammatory cells in gut mucosa compared to the PBS control group and low-does lysates, which suggested that the gastrointestinal administration of excessive and long-termed lysates could increase a local inflammation response in gut. The AOM/DSS CRC model is colitis-associated carcinoma, so we choose low dose lysates (lysates of 5 × 10^5^ CFU *L. acidophilus*) combined with CTLA-4 antibody. Moreover, we founded that lysates of *L. acidophilus* promote multiplex antitumor immune effects of CTLA-4 mAb in the CRC mouse model by decreasing the numbers of Treg and M2 cells, and increasing T cells and cytokine levels (Granzyme B, TNF-α and IFN-γ). Such lysates may be suitable “cancer-immunotherapy enhancers” for CTLA-4 Ab, both owing to their inhibition of the growth of abnormal bacteria in CRC mice and synergy with the Toll-like receptor (TLR) signaling pathway.

Frankly, the current study has several limitations. First, as heterologous antigens, the administration of *Lactobacillus acidophilus* lysates undoubtedly can trigger many types of innate and adaptive immune responses in healthy and CRC model mice. Due to the lack of well-established methods to acquire cytotoxic T cells and antibodies against the lysates antigens, here we only discuss the synergistic anti-tumor effects of innate immunity leaded by the lysates and anti-CTLA-4 antibodies. Second, although the inflammatory bowel disease (IBD) development) development of cancer in this AOM/DSS model closely mirrors the pattern seen in humans and accurately recapitulate the pathogenesis observed in human CRC, the IBD-related colon cancer in humans represents a relatively rare occurrence (1% of cases). Additionally, AOM-induced tumors often lack mucosal invasiveness^37^. So, the synergistic anti-tumor effects of *Lactobacillus acidophilus* lysates combined with CTLA-4-blocking antibodies need to be further confirmed in multiple CRC tumor models.

In conclusion, here, we show that lysates of *L. acidophilus* are promising enhancers of cancer immunotherapy for CRC. CTLA-4 blocking antibodies in combination with the present lysates may be of importance for the development of new therapeutic strategies against CRC to be tested in clinical trials.

### Ethics approval and consent to participate

All mouse work was reviewed and approved by the Ethics Committee for Animal Research of Nanjing Medical University and the Healthcare Animal Care and Use Committees of Nanjing First Hospital.

## Supplementary information

Supplementary information

## Data Availability

The datasets used and analyzed during the current study are available from the corresponding author on reasonable request.
