# Supplementary material for: Lysates of Lactobacillus acidophilus combined with CTLA-4-blocking antibodies enhance antitumor immunity in a mouse colon cancer model
Source: Sci Rep. 2019 Dec 27;9:20128. doi: 10.1038/s41598-019-56661-y (PMC6934597; doi:10.1038/s41598-019-56661-y)
Supplement: Supplementary file 1 — Supplementary information [file 41598_2019_56661_MOESM1_ESM.pdf]

Lysates of *Lactobacillus acidophilus* combined with CTLA-4-blocking antibodies enhance antitumor immunity in a mouse colon cancer model

Qian Zhuo[1,2#], Bohai Yu [3#], Jing Zhou[1], Jingyun Zhang[1], Runling Zhang[4], Jingyan Xie[1], Qingling Wang[2*] and Shuli Zhao[1*]

[1]General Clinical Research Center, Nanjing First Hospital, Nanjing Medical University, Nanjing, 210006, China

[2] Department of Pathology, Xuzhou Medical College, Xuzhou, Jiangsu, 221004, China

[3]Medical Laboratory Department, Shenzhen Hospital of Guangzhou University of Chinese Medicine, Shenzhen, Guangdong, 518034, China

[4]University of Chinese Academy of Sciences Shenzhen Hospital, Shenzhen, Guangdong, 518106, China

[#]Qian Zhuo and Bohai Yu contributed equally to this work.

***Correspondence to**:

Nanjing First Hospital, No. 68 Changle Road, Nanjing 210006, China

Tel & Fax: +86-25-522887003

Corresponding author:

Shuli Zhao, email: shulizhao79@163.com

Qingling Wang, email: qlwang@xzhmu.edu.cn

Running Title: *L. acidophilus* lysates enhances cancer immunotherapy

Supplementary

Table S1. Lymphocytes subsets in peripheral blood of mice used in the safety evaluation study (Mean±SD, %)

Parameter	PBS group	Low-does group	High-does group
CD3+CD8+ T cell	14.4±2.7	13.8±5.9	15.4±4.5
CD3+CD4+ T cell	19.6±7.8	23.7±6.9	22.8±5.6
CD4+CD25+Foxp3+ Treg cell	2.4±1.5	3.6±0.7	3.3±1.5
DX5+ NK cell	14.4±9.3	15.6±4.5	11.3±5.1
CD19+ B cell	24.7±9.9	28.5±6.5	26.2±3.7

Table S2. Lymphocytes subsets in mesenteric lymph nodes of mice used in the safety evaluation study (Mean±SD, %)

Parameter	PBS group	Low-does group	High-does group
CD3+CD8+ T cell	16.6±4.7	15.8±6.9	17.1±3.8
CD3+CD4+ T cell	24.6±10.8	27.7±7.1	29.9±6.1
CD4+CD25+Foxp3+ Treg cell	3.1±2.4	2.9±2.2	3.9±2.7
DX5+ NK cell	15.8±6.5	15.6±4.7	18.4±4.3
CD19+ B cell	34.1±10.1	32.5±8.2	30.5±7.2

Table S3. Body weight of mice in safety evaluation study (Mean±SD, g)

Days	PBS group	Low-does	High-does
D1	22.8±2.2	21.7±2.4	22.1±2.7
D8	23.3±2.8	23.7±2.5	24.7±2.4
D15	25.2±3.4	24.9±3.3	25.2±2.4
D22	26.5±3.8	25.9±3.6	25.7±3.7
D29	26.6±4.0	26.5±3.1	27.0±3.4

Table S4. The primers used for qPCR assays

Table S4. The primers used for qPCR assays

Genes	Forward primer	Reverse primer
Actin	CGTGAAAAGATGACCCAGATCA	CACAGCCTGGATGGCTACGT
IL-2	CCTGAGCAGGATGGAGAATTACA	TCCAGAACATGCCGCAGAG
IL-10	CCAGAGCCACATGCTCCTAGA	GGTCCTTTGTTTGAAAGAAAGTCTTC
IFN-γ	CAGCAACAGCAAGGCGAAA	CTGGACCTGTGGGTTGTTGAC
TNF- α	CATCTTCTCAAAATTCGAGTGACAA	CCAGCTGCTCCTCCACTTG

Figure S1.

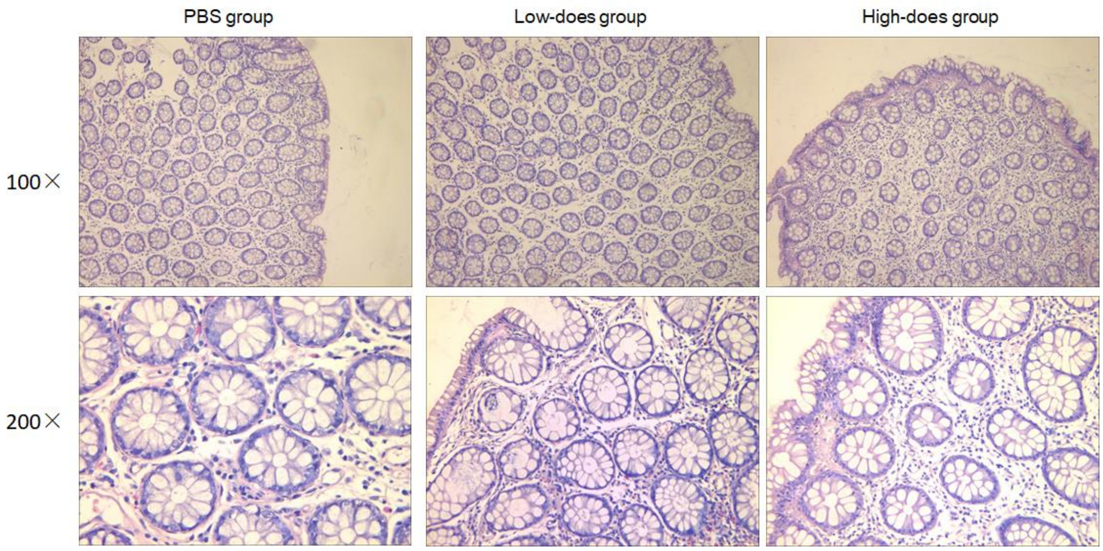

Representative H&E-stained images of colic tissues from mice in safety evaluation study of *L. acidophilus* lysates. Images were acquired using the Olympus BX50 microscope (magnification, × 100 and × 200).
